# Acoustic Multi-Detection of Gliadin Using QCM Crystals Patterned with Controlled Sectors of TEM Grid and Annealed Nanoislands on Gold Electrode

**DOI:** 10.3390/nano10040790

**Published:** 2020-04-20

**Authors:** Giuliocesare Casari Bariani, Lan Zhou, Simone Poggesi, Rakesh Mittapalli, Marisa Manzano, Rodica Elena Ionescu

**Affiliations:** 1 Laboratoire Lumière, Nanomatériaux et Nanotechnologies – L2n, Université de Technologie de Troyes, CNRS ERL 7004, 12 rue Marie Curie, CS 42060, 10004 Troyes CEDEX, France; casaribariani.giuliocesare@spes.uniud.it (G.C.B.); lan.zhou@utt.fr (L.Z.); poggesi.simone@spes.uniud.it (S.P.); rakesh.mittapalli@utt.fr (R.M.); 2 Dipartimento di Scienze Agroalimentari, Ambientali e Animali (DI4A), Università degli Studi di Udine, Via Sondrio 2/A, 33,100 Udine, Italy; marisa.manzano@uniud.it

**Keywords:** patterning nanoislands, annealed gold films, annealed quartz, Q-TEM and QCM-color crystals, multi-sensing platforms, gliadin

## Abstract

Celiac diseases are a group of gluten ingestion-correlated pathologies that are widespread and, in some cases, very dangerous for human health. The only effective treatment is the elimination of gluten from the diet throughout life. Nowadays, the food industries are very interested in cheap, easy-to-handle methods for detecting gluten in food, in order to provide their consumers with safe and high-quality food. Here, for the first time, the manufacture of controlled micropatterns of annealed gold nanoislands (AuNIs) on a single QCM crystal (QCM-color) and their biofunctionalization for the specific detection of traces of gliadin is reported. In addition, the modified quartz crystal with a TEM grid and 30 nm Au (Q-TEM grid crystal) is proposed as an acoustic sensitive biosensing platform for the rapid screening of the gliadin content in real food products.

## 1. Introduction

Cereals such as wheat, rye, barley, and oats provide carbohydrate, vitamins, minerals, soluble and insoluble fibers, and proteins. However, they represent also an allergenic source for some people. The 80% of the proteins present in cereals (8–15%) is represented by gliadin and glutenin, among which gliadin is the major allergen. In fact, it is responsible for three different gluten-related pathologies: gluten allergy, celiac disease, and gluten sensitivity [1].

Nowadays, the scientific community is focused on the individuation of fast and cheap methods for gluten detection in food products. Regulation (EC) No. 41/2009 of the European Union fixes the limit of gluten at <20 mg/kg (20 ppm) for all foodstuffs labeled as “*gluten free*”, and about 20–100 mg/kg (100 ppm) for the label “*very low gluten*” [2].

Several methodologies for gluten detection have been realized in last few decades: immunological techniques [3,4]; mass spectrometric and chromatographic techniques [5]; Western blot and proteomic techniques [6,7]; quantitative real-time polymerase chain reaction (Q-PCR) [8]; aptamers against gliadin, as alternative to antibodies [9].

The development of new sensitive methods is a crucial thematic in the food industry. Biosensor-based detections are becoming very widespread, since they are usually cheaper and more sensitive than the common methods. A biosensor is an analytical tool that uses a biological recognition element to target various molecules. They are often based on nanotechnological devices that use noble metals for their applications [10]. The use of metal nanoparticles, especially gold (AuNPs), in the field of biomatter interactions attracts considerable interest for daily-based applications. AuNPs can serve as miniaturized platforms that are ideal for the development of ultrasensitive bioassays [11,12]. Thanks to NPs’ physical properties, they can be distributed on different supports, in order to modify the surface in terms of roughness, thickness, area, and distribution, modulating the support’s sensitivity [13].

Among a large variety of biosensors, quartz crystal microbalance (QCM) is showing powerful applications in the biodetection of food toxins [14], micro-organism metabolites [15,16], environmental pollutants [17,18], agricultural pesticides [19], and also in clinical diagnosis [20]. It consists of a thin piezoelectric plate with gold electrodes on both sides. Due to the piezoelectric effect, if an AC voltage is applied across the electrodes, a crystal oscillation is induced. Piezoelectric devices can measure very low masses by measuring the change in frequency of the quartz crystal.

Many studies have reported biosensors-based nanomaterials with gold nanoparticles (AuNPs) as ultrasensitive sensing platforms. Such low-cost sensors provide a high surface area and easy process [21]. By modifying the surface of the quartz crystal with AuNPs, it is possible to change the frequency shift, increasing the sensitivity of the tool [22]; in fact, through increasing of the number of binding sites for the targeted antigen (gliadin in this case), the sensitivity of the system is increased. However, the analytical performances of the biosensor can still be sensitive to the sample preparation step and to the stability of AuNPs colloidal solution deposed on the quartz surface. Recently, monoclonal and polyclonal antibodies conjugated to colloidal AuNPs have been used for the real-time acoustic detection of *Campylobacter jejuni* cells with a positive increase on the frequency direct proportional to cell concentration [23].

Herein, simple and reproducible protocols are proposed to perform a robust gold-structuration of commercial QCM quartz crystals (named S-QCM) following two approaches: (i) TEM grids patterning (named Q-TEM grid crystal) and (ii) the annealing of home-evaporated gold thin films to form gold nanoislands (AuNIs) on a gold electrode’s crystal (named QCM-color crystal). To evaluate the role of AuNIs in modulating the acoustic sensitivity, different gold thicknesses were tested; after labeling the nanostructured crystals with an anti-gliadin antibody, they were used for acoustic biosensing with different concentrations of gliadin.

The present work aims to evaluate the sensing performances, in terms of changes in frequency (ΔF), of QCM crystals after their modification with either TEM grids or gold nanoislands (AuNIs), in comparison to standard non-structured quartz S-QCM crystal. Therefore, the authors consider that the existence of different sensitivity areas can be equivalent to the existing gauges on certain measuring instruments. If it is not necessary to accurately detect the target, but only to control its presence or absence in a sample, with an indication of the interval, a sample drop will be deposited first in the less sensitive area toward the more sensitive areas until a positive response will be recorded. In addition, Q-TEM grid and QCM-color crystals can be used for the direct biofunctionalization of nanoislands structured a gold electrode with no need of organic solvents. The annealed crystals are stable at room temperature and aqueous conditions.

## 2. Materials and Methods

### 2.1. Chemicals

Ethanol 70% was obtained from Sigma-Aldrich (St. Louis, MO, USA). 11-Mercaptoundecanoic acid (11-MUA), gliadin from wheat, polyclonal rabbit an anti-gliadin antibody, monoclonal anti-BSA (bovine serum albumin) antibody produced in mouse, 1-ethyl-3-[3-dimethylaminopropyl]-carbodiimide hydrochloride (EDC), and N-hydroxy succinimide (NHS) were purchased from Sigma-Aldrich (Schnelldorf, Germany). Sterile double-distilled water (named S-ddwater) (18.2 MΩ*cm) was produced by a Millipore Milli-Q water purification system (Molsheim, France).

All the chemicals were of analytical grade and used as received without further treatment. Different “gluten-free” and “might contain gluten” labeled food products have been purchased from a local supplier: biscuits, snacks, and food supplement.

### 2.2. Instruments

All acoustic measurements were carried out with a QCM200 quartz crystal microbalance (Stanford Research Systems, CA, USA) including a crystal oscillator QCM25 and a crystal holder that can accommodate a quartz crystal of 5 MHz (2.54 cm) AT-cut with gold electrodes on both sides (Cr/Au) (Stanford Research Systems, CA, USA).

A gold evaporation step was performed with the evaporator Plassys MEB 400 (Plassys, Bestek, France) to fabricate Q-TEM grid and QCM-color quartz crystals.

S-ddwater was obtained after the sterilization of distillated water with a Tuttnauer Autoclave Steam Sterilizer 2540ML (Tuttnauer, Villenoy, France). A VWR DRY-Line dry DL 53 oven was used for drying processes, while the biofunctionalization steps were performed under a biological hood MSC 1,2 ADV (Thermo-scientific, Illkirch Cedex, France). Standard QCM (S-QCM) crystals were cleaned with S-ddwater and detergent (Decon 90) solution (2:8, *v*/*v* ratio) at 50 °C for 15 min in an ultrasonic bath (Elmasonic S30H), according to Kun et al. [24].

QCM crystals were characterized with a scanning electron microscope (SEM) (FEG-SU8030, Tokyo, Japan).

The nanoparticle size distribution and background proportion were analyzed using the Public Domain ImageJ software developed by the National Institutes of Health based on the SEM images, The “Threshold” function was used to obtain the proportion of background in percentage for uncovered quartz by annealed gold nanoparticles, while the function “Analyze Particles” was used to analyze the size of nanoparticles.

### 2.3. Preparation of Gliadin Concentrations Using Commercial Powder

A stock solution of gliadin (80 g/L ethanol/S-ddwater in 6:4 *v*/*v* ratio) was prepared by weighing 0.080 g ± 0.001 g of gliadin and dissolving in a solution containing 0.6 mL of ethanol and 0.4 mL of S-ddwater. Five gliadin concentrations were freshly prepared for each set of acoustic sensing experiments by diluting the stock solution with S-ddwater: 1 mg/L (1 ppm), 10^−1^ mg/L (0.1 ppm), 10^−2^ mg/L (0.01 ppm), 10^−3^ mg/L (0.001 ppm), and 10^−4^ mg/L (0.0001 ppm).

### 2.4. Extraction of Gliadin from Commercial Foods

Five food samples were purchased from a local supplier with the gliadin content mentioned on the package label as follows: (i) vegan food supplement (gluten-free); (ii) corn chilly snack (gluten-free); (iii) rice snack (might contain gluten); (iv) rice noodles (might contain gluten); and (v) chocolate biscuits (gluten-free). Further, the gliadin was extracted according to the method proposed by Chu et al., (2012), with some modifications: 1 g of each food was weighed and diluted in 10 mL of S-ddwater, mixed at room temperature for 6 h, centrifuged at 5000 rpm for 10 min, collecting the pellets that were further resuspended in 10 mL of ethanol/S-ddwater solution (6:4, *v*/*v* ratio) to obtain a stock solution after used in QCM sensing tests.

### 2.5. Modification of S-QCM Crystals with TEM-Grid and Annealed Nanostructures

**Figure 1:**
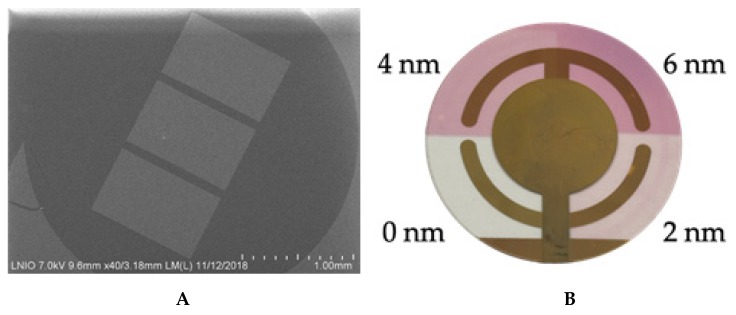
No annealed Q-TEM grid crystal with gold electrode modified with 30 nm Au evaporated through a TEM grid with three slots (**A**); Annealed QCM-color crystal modified with three sectors of Au nanoislands on a gold electrode and with nanoparticles on quartz as a function of the thickness of evaporated gold (2 nm, 4 nm, and 6 nm) and with one quartz sector without metal modification (as received commercially) (**B**).

S-quartz crystals were modified with either 30 nm or 50 nm gold, evaporated through a TEM-grid mask (Q-TEM grid) or with annealed gold nanoparticles on glass/nanoislands on gold electrode and size-dependent of the initial evaporated gold (QCM-color) (Figure 1).

#### 2.5.1. Fabrication of Q-TEM Grid Crystals

Crystals were first rinsed and washed in an ultrasonic bath with S-ddwater at 50 °C for 5 min, washed with S-ddwater, followed by drying under nitrogen stream, and deposition on a hot plate at 100 °C for 10 min to completely dry the surface.

Gold thin films (30 nm or 50 nm) were evaporated at 25 °C by using 1 × 10^−5^ Torr pressure with an evaporation rate of 0.2 nm/s and with no further annealing (Figure 1A) through TEM grids of 3 slots over two individual QCM crystals (the rest of the quartz surfaces were protected with regular scotch pieces).

#### 2.5.2. Fabrication of QCM-Color Crystals with Sectors of Annealed Gold Nano-Islands

A QCM-color crystal (Figure 1B) was fabricated by using three glass square coverslips 22 × 22 mm (Carl Roth GmbH + Co.KG, Germany) to protect different quartz sectors to control gold coating, and the whole crystal system was fixed on a metallic support in the evaporation chamber. In this work, three consecutive evaporations of 2 nm on each sector have been performed. To obtain 4 nm and 6 nm gold coatings on well-defined crystal sectors, coverslips were used as masks. The evaporation was performed at 25 °C by using 1 × 10^−5^ Torr pressure with an evaporation rate of 0.03 nm/s and with no further annealing (Figure 1B). The resulted gold-modified quartz crystal was heated on a preheated hot plate (Thermo Fisher Scientific, USA) for different periods of time and temperatures to avoid the crystals breaking: (a) 5 min at 40 °C, (b) 5 min at 60 °C, (c) 5 min at 90 °C, (d) 10 min at 150 °C, (e) 10 min at 200 °C, (f) 10 min at 250 °C, (g) 20 min at 350 °C, (h) 20 min at 450 °C, and (i) 6 h at 550 °C, respectively.

### 2.6. Cleaning and Thiol-Functionalization of S-QCM, Q-TEM grid and QCM-Color Quartz Crystals

S-QCM, Q-TEM grid, and QCM-color quartz crystals were firstly washed with an ethanol solution (70%) at 40 °C in an ultrasonic bath for 20 min, and after S-ddwater for 10 min in an ultrasonic bath at 40 °C, to avoid the ethanol traces on the crystals’ surface. The resulting cleaned crystals were dried at 50 °C for 20 min. Subsequently, the crystals were thiolate by overnight dipping in 5 mL of 11-MUA (10^−3^ M) in ethanol solution (70%) at room temperature. The day after, the crystals were abundantly rinsed with ethanol and S-ddwater and dried under a biological hood.

### 2.7. Biofunctionalization of S-QCM, Q-TEM-grid, and QCM Color Crystals with Specific or Non-Specific an Anti-Gliadin Antibody

**Figure 2:**
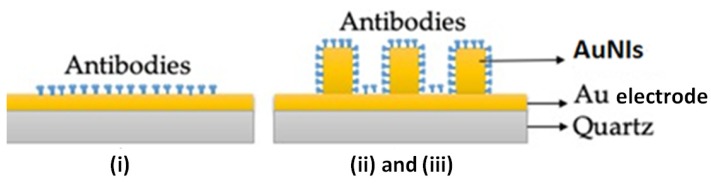
A simple representation of the (bio)functionalization of an Au electrode of quartz crystal with antibodies when using: (i) S-QCM crystal; (ii and iii) Q-TEM grid and QCM-color crystals (AuNIs: gold nanoislands).

The thiolated QCM crystals were activated by their dipping in 5 mL of EDC/NHS 0.4/0.1 mM solution for 30 min at room temperature, followed by S-ddwater washing and drying under the biological hood. Crystals were after biofunctionalized with an anti-gliadin antibody (Figure 2), by pouring on top of the gold chip side 40 μL of antibody at 100 μg/mL in S-ddwater and incubated overnight at 4 °C.

To validate the specific detection of different gliadin concentrations, control experiments were performed on an anti-BSA antibody biofunctionalized S-QCM and Q-TEM (30 nm Au) crystals. Before the utilization, all QCM crystals were washed twice with sterile S-ddwater and incubated with anti-BSA antibody (40 μL, 125 μg/mL) in S-ddwater for overnight at 4 °C. The day after, the QCM crystals were abundantly rinsed with S-ddwater, dried under a biological hood, and used for the acoustic detection of different gliadin concentrations.

For S-QCM crystals, two sets of experiments were performed: one with an anti-gliadin antibodies (specific)-labeled crystal and one with anti-BSA antibodies (non-specific)-labeled crystal.

### 2.8. Step-By-Step Acoustic Measurements

For the experiments, the antibody biofunctionalized QCM crystals were fixed on the crystal holder of the acoustic balance, and the absolute frequency baseline of the crystal in air was stabilized. After stabilization, a drop of 10 μL S-ddwater was deposed as the reference signal. After 20 min, the S-ddwater was collected, and the crystal was left dry to stabilize the absolute frequency for 15 min.

Two sets of experiments were performed with S-QCM and Q-TEM grid crystals: first with a specific an anti-gliadin antibody-labeled crystal and second with a non-specific BSA antibody-labeled crystal. The standard gliadin solutions were tested starting from the lowest analyte concentration (10^−4^ mg/L), to the highest 1 mg/L). Each drop of 10 μL gliadin solution was deposed on the crystal for 40 min, then collected, and the crystal was rinsed twice with S-ddwater before the deposition of 10 μL of a different gliadin concentration (10^−3^ mg/L) on the QCM crystal for 40 min. Each concentration was collected after 40 min deposition, and the crystal was rinsed twice, before any new deposition with 10 μL S-ddwater. Twenty minutes were necessary for the stabilization of the absolute frequency before the analyses.

A Q-TEM grid crystal was used for testing the gliadin content extracted from five food products; thus, 0.6 mL of each independent stock solution were carefully mixed with 0.4 mL of S-ddwater and used for the collection of 4 μL of gliadin solution that were shortly placed on the antibody modified QCM crystal. Further, the crystal was twice washed with S-ddwater from the excess of a specific an anti-gliadin concentration, dried, and used for the acoustic investigations.

For the QCM-color crystal, a specific procedure was used. The four nanostructured zones of the QCM crystal (0 nm, 2 nm, 4 nm, and 6 nm Au) were exposed to successive depositions and collections of 4 drops of S-ddwater and aqueous gliadin solution of 10^−4^ mg/L and 10^−3^ mg/L. Before dropping liquids, a dry crystal was mounted on a plastic holder and exposed to air to achieve frequency stabilization for 20 min with the microbalance switched on.

## 3. Results and Discussion

### 3.1. SEM Characterization of S-QCM, Q-TEM Grid, and QCM-Color Crystals

**Figure 3:**
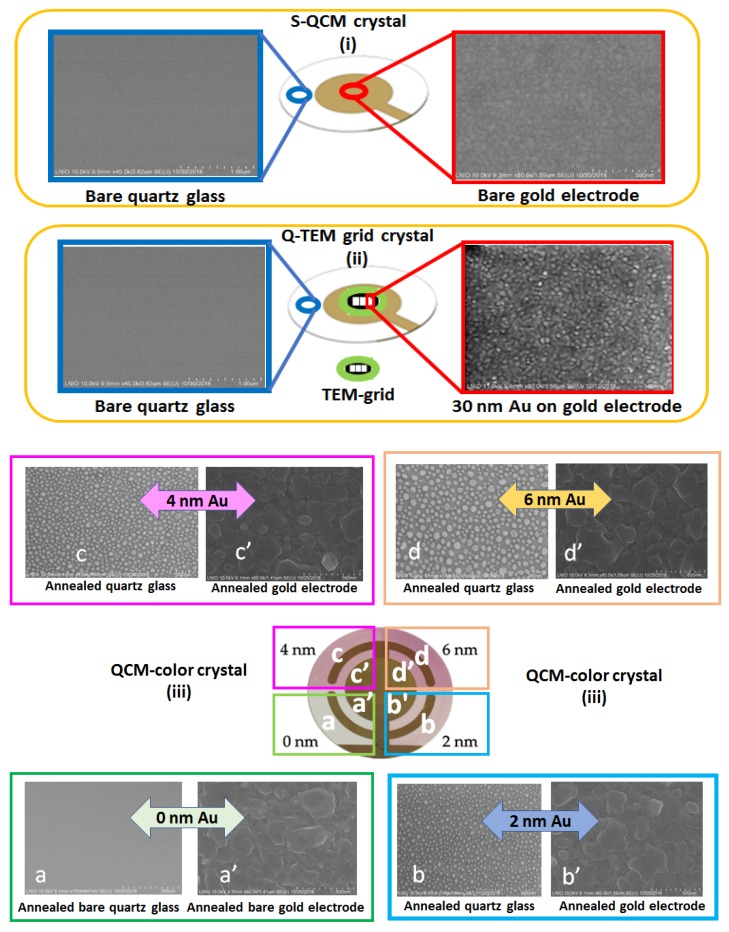
SEM images of three types of QCM crystals used for the (bio)functionalization and detection of gliadin (i) bare S-QCM crystal, S-standard; (ii) no annealed Q-TEM grid crystal with gold electrode modified with 30 nm Au evaporated through a TEM-grid; (iii) annealed QCM-color crystal modified with different areas of controlled sectors of nanoparticles on quartz and nanoislands on the gold electrode as a function of the thickness of evaporated gold films (a, b, c, d—SEM images of the quartz; a’, b’, c’, d’—SEM images of the gold electrode).

Scanning electron microscopy characterization of S-QCM, Q-TEM, and QCM-color crystals is illustrated in Figure 3 for the gold electrode and outside the electrode, which is the transparent part of crystals.

Around the gold electrodes for S-QCM and Q-TEM crystals as well as for the QCM-color crystal with 0 nm Au, a homogeneous structure is observed without granulation, corresponding to the expected structure of a bare quartz crystal observed also by Niu et al., 2018 [25]. The gold electrode of the S-QCM quartz consists of regular 50-nm sized grains, well connected without porosity. However, the deposition of 30 nm Au to obtain Q-TEM crystal induced a visible structuration of the gold electrode with a regular height modification of the former layer.

The annealing treatment of the Au-deposited layer (2 nm, 4 nm, and 6 nm) on three sectors of the QCM-color crystals leads to the formation of nanoparticles outside the former electrode (bare transparent quartz) and of nanoislands on the gold electrode. The difference between the observed morphologies (nanoparticles versus nanoislands) is probably due to the difference between the expansion thermal coefficients more important for Au-deposited layer/quartz interface than for gold electrode/Au-deposited layer interface. Thus, the morphology of annealed gold electrode coated with 6 nm Au shows the highest granulation effect when compared with those of 4 nm Au and 2 nm Au, respectively.

**Figure 4:**
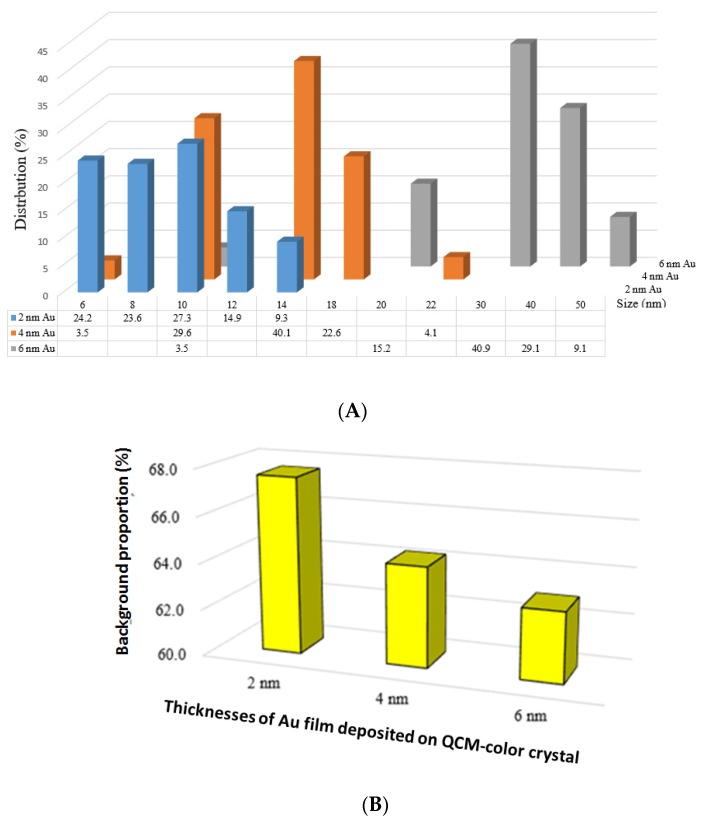
Analysis of gold nanoparticles size distribution using the SEM images from Figure 3. (**A**) for different evaporated gold thickness and (**B**) the background proportion of uncovered crystal by gold nanoparticles after annealing on a preheated hot plate for about 7 h.

Moreover, Figure 4A shows the size distribution of nanoparticles formed on quartz transparent glass after annealing as a function of the evaporated gold thicknesses (2 nm, 4 nm, and 6 nm, respectively) when using the SEM images of Figure 3. As expected, the nanoparticles sizes increase with the increases of the evaporated gold layer from 2 nm Au (6 nm to 14 nm), to 4 nm Au (6 nm to 22 nm) and to 6 nm (10 nm to 50 nm), and it is in good agreement with Figure 4B, which depicts the background proportion for each gold thickness: 2 nm (67.3%), >4 nm (63.9%), and >6 nm (62.5%).

### 3.2. Acoustic Gliadin Detection Using S-QCM and Q-TEM Grid Crystals

**Figure 5:**
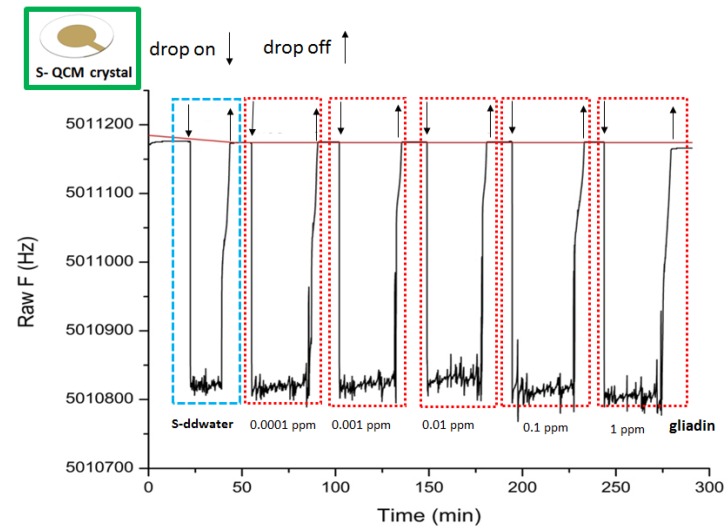
Sensorgram of S-QCM crystal biofunctionalized with an anti-gliadin antibody and exposed to different concentrations of gliadin.

**Figure 6:**
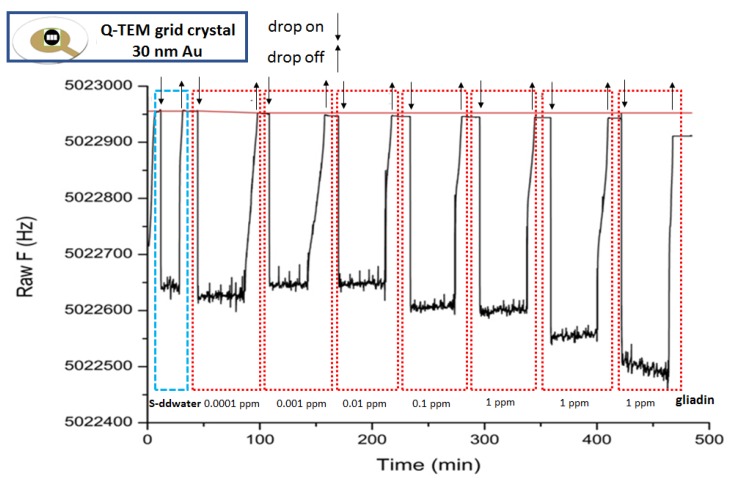
Sensorgram of Q-TEM grid (30 nm Au) crystal biofunctionalized with an anti-gliadin antibody and exposed to different concentrations of gliadin.

**Figure 7:**
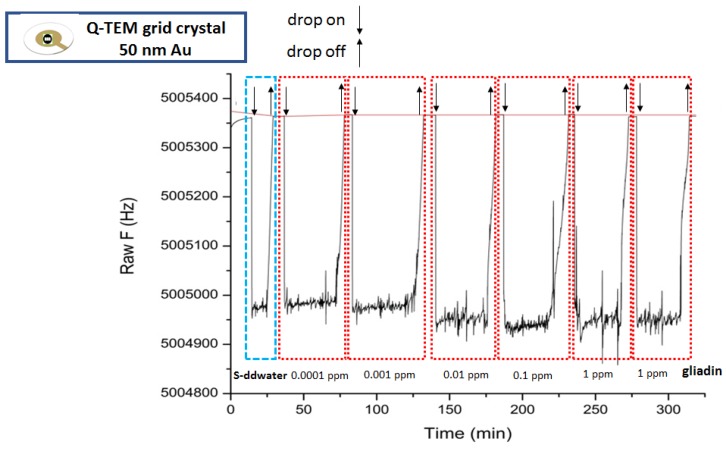
Sensorgram of Q-TEM grid (50 nm Au) crystal biofunctionalized with an anti-gliadin antibody and exposed to different concentrations of gliadin.

Figure 5, Figure 6 and Figure 7 illustrate the sensorgrams obtained for S-QCM, Q-TEM grid (30 nm Au), and Q-TEM grid (50 nm Au) crystals when drops of gliadin (10 µL) at concentrations ranging from 10^−4^ mg/L (0.0001 ppm) to 1 mg/L (1 ppm) are deposited.

**Figure 8:**
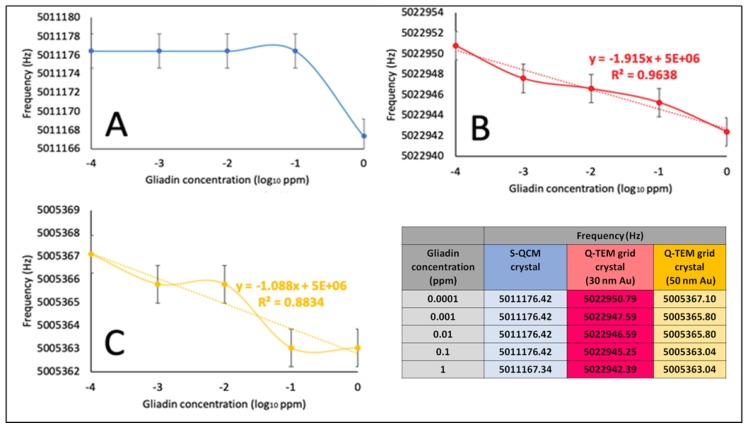
Dose/response curves of the immune-acoustic gliadin detection on: (**A**) S-QCM crystal; (**B**) Q-TEM grid (30 nm Au) and (**C**) Q-TEM grid (50 nm Au) crystals.

Frequencies were extracted at stabilization of the signal after drops collection, and plotted as a function of the concentration of gliadin (Figure 8), allowing the construction of dose/response curves.

For S-QCM (Figure 8A), the frequency keeps constant from 0.0001 ppm to 0.1 ppm and starts to decrease for 1 ppm. This indicates that the limit of detection is higher than 0.1 ppm. In the case of the two Q-TEM grid crystals (Figure 8B,C), a decrease of the frequency is observed from 0.0001 ppm to 1 ppm, indicating that the limit of detection is equal to or lower to 0.0001 ppm. Moreover, the response is more linear for the Q-TEM grid crystal with 30 nm Au (*R*^2^ = 0.9636) than for the Q-TEM grid crystal with 50 nm Au (0.8834). In addition, the Q-TEM grid crystal with 30 nm appears to be more sensitive for the detection of gliadin traces. These results prove the beneficial impact of the gold layer deposited through a TEM grid by improving the limit of detection of gliadin and the dependence of the response of quartz on the thickness of gold.

### 3.3. Acoustic Gliadin Detection Using QCM-Color Crystal with Different Sectors: 0 nm, 2 nm, 4 nm, and 6 nm Au

**Figure 9:**
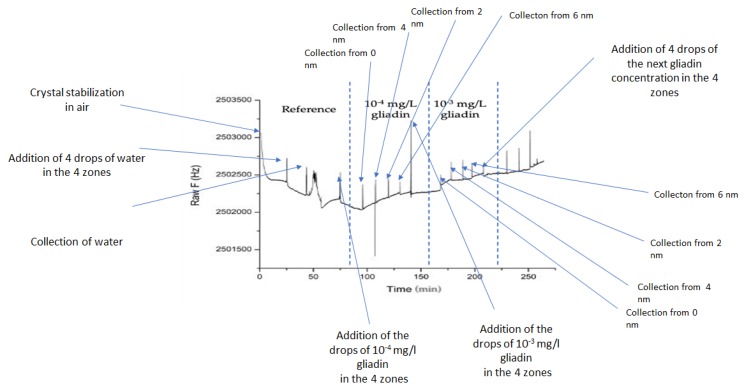
Acoustic sensorgram of QCM-color crystal biofunctionalized with an anti-gliadin antibody and exposed to gliadin concentrations.

Figure 9 depicts the frequency variation of an annealed QCM crystal (QCM-Color) biofunctionalized with an anti-gliadin antibody following the procedure described in Section 2.7. Four drops of S-ddwater induced a decrease in the resonance frequency of 182 Hz. However, when the water drops were collected, an increase in the resonance frequency was observed. These results are good in agreement with Sauerbrey’s law. In addition, when the 4 drops of sterile S-ddwater were completely collected, the resonance frequency of the QCM crystal was lower than that measured at the beginning of the acoustic experiment. This can be explained by the inevitable presence of water impurities on the crystal surface. Subsequently, the 4 drops of 10^−4^ mg/L gliadin solution cause a change in frequency similar to that observed after water drops (−133 Hz). As expected, the collection of droplets induces an increase in the resonance frequency.

The difference between the resonance frequency measured before the addition of the drops and the last one measured after the collection of the 4 drops (+93 Hz) can be attributed to the recognition events between the gliadin target and an anti-gliadin antibody modified QCM crystal. This difference is positive, highlighting an anti-Sauerbrey behavior that is probably due to the particular bio-interaction between the target and the probe, which is most probably due to the presence of nanostructures. Similar frequency behavior has already been reported [26]. In addition, the deposition of the 4 drops of gliadin solution of 10^−3^ mg/L on 4 zones presents a major difference compared to the two previous experiments. The frequency remains almost constant before and after the deposition of drops instead of its decrease. Such a result shows that the sensibility of the quartz is not uniform and depends on the area where the dropping was carried out. Therefore, before using a multi-zone QCM quartz, it is necessary to map the areas that have the same sensitivity. This problem can be solved by measuring the decrease of the frequency resonance after 4 drops of water are deposited on different areas of the crystal. These are the first results that show that an annealed nanostructured quartz can be used to detect gliadin.

### 3.4. Control Experiments—In the Presence of Non-Specific an Anti-Gliadin Antibody

**Figure 10:**
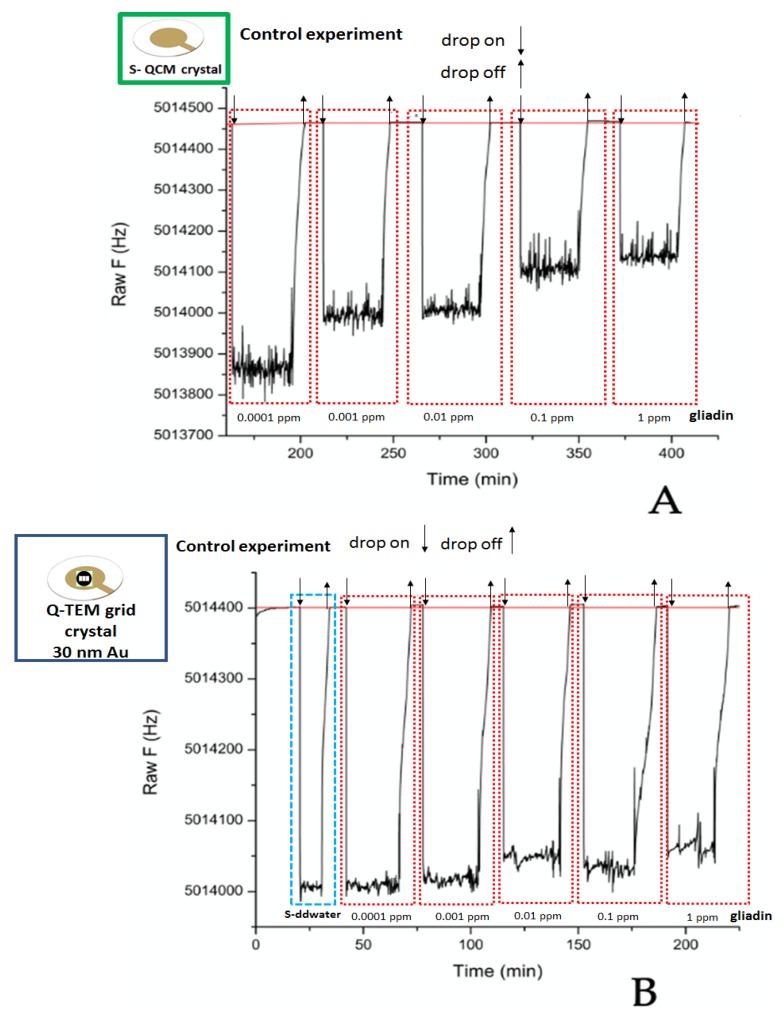
Control experiments: Sensorgrams of S-QCM (**A**) and Q-TEM grid (30 nm Au) (**B**) crystals biofunctionalized with an anti-gliadin antibody and exposed to different concentrations of gliadin.

S-QCM (Figure 10A) and Q-TEM grid (30 nm Au) crystals (Figure 10B) were biofunctionalized with non-specific an anti-gliadin antibody: namely, anti-BSA antibody. As expected, for both crystal configurations, there was no logical shift of the frequency in the presence of five gliadin concentrations (0.0001 ppm; 0.001 ppm; 0.01 pp; 0.1 ppm; and 1 ppm). These results demonstrate the selectivity of the constructed gliadin immunosensors.

### 3.5. Acoustic Detection of Gliadin Traces in Food Using Q-TEM Grid Crystal (30 nm Au)

**Figure 11:**
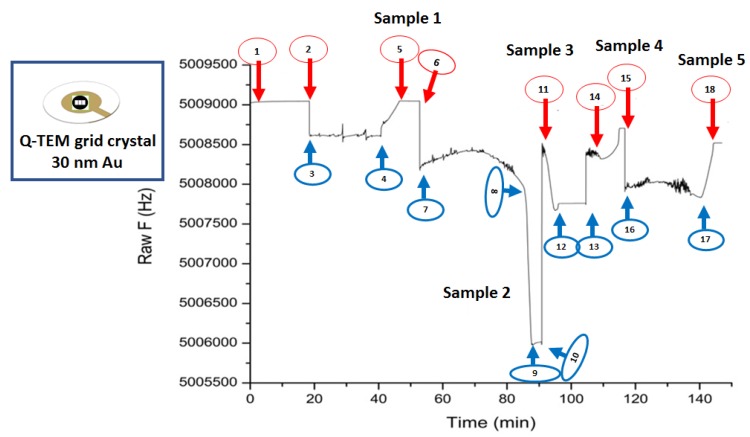
Screening the presence of gliadin in food products using Q-TEM grid (30 nm Au) crystal biofunctionalized with an an anti-gliadin antibody combined by the drop-deposition procedure of aqueous solutions of real food that might contain gliadin traces (4 µL): (1) vegan food supplement (gluten-free labeled), (2) corn chilly snack (gluten-free labeled), (3) rice snack (might contain gluten labeled), (4) rice noodles (might contain gluten labeled), (5) chocolate biscuits (gluten-free labeled). Step-by-step measurements: (Crystal mounted in the QCM – 1; drop deposition – 2 (S-ddwater), 6 (sample 1), 8 (sample 2), 11 (sample 3), 14 (sample 4), 15 (sample 5); QCM response after deposition – 3 (S-ddwater), 7 (sample 1), 9 (sample 2), 12 (sample 3), close to 14 (sample 4), 16 (sample 5); drop collection – 4 (S-ddwater), closed to 8 (collection impossible, sample 1 dried), 10 (sample 2), 13 (sample 3), closed to 14 (sample 4), 17 (sample 5); QCM response after collection – 5 (S-ddwater), impossible for sample 1, 11 (sample 2), 13 (sample 3), 15 (sample 4), 18 (sample 5).

An acoustic sensorgram (Figure 11) for five food samples was recorded with Q-TEM grid (30 nm Au) crystal biofunctionalized with an an anti-gliadin antibody.

The whole procedure (deposition, stabilization, collection, stabilization) has been successfully completed for S-ddwater (2, 3, 4, 5), sample 2 (8, 9, 10, 11), sample 3 (11, 12, 13, 14), and sample 5 (15, 16, 17, 18). Problems were encountered with sample 1 and 4. Sample 1 dried on the crystal surface during the test was therefore uncollectable. The dropping of sample 4 on the surface quartz failed, resulting in a small frequency shift. Consequently, the only exploitable results are those obtained on S-ddwater and samples 2, 3 and 5.

In the case of S-ddwater, as expected, the deposition of the drop induced a decrease in the resonance frequency, while its collection induced an increase. The increase in frequency value exactly compensated for the decrease in frequency, and therefore, the final offset of the resonant frequency was zero, because no bonding occurred. For sample 2, a positive shift is observed while a negative frequency value is observed for sample 5 and no significant change for sample 3.

In the presence of sample 3 (rice snack), the QCM measurements indicate that there is no gliadin, while the food packaging indicates that it may contain gluten.

As for sample 2 (corn chilly snack), the label indicates that the food is gluten-free, while a positive frequency change was observed. This positive evolution is questionable, because the previous experiment has shown that the behavior of the Q-TEM grid crystal follows the Sauerbrey law.

Finally, for sample 5 (chocolate biscuits), a negative shift in frequency for gliadin was recorded, although the absence of gluten is indicated on the food label.

These very primary results show the limits of acoustic sensing to estimate the presence of gliadin in complex foods. In fact, the extraction of gliadin was not selective enough to avoid the extraction of other food components such as fats and oils, causing high levels of interference. Thus, at the end of the experiment, the crystal was very dirty and difficult to rinse.

## 4. Conclusions

Acoustic detection using home-prepared Q-TEM grid (30 nm Au) and QCM-color crystals have proved to be efficient platforms for the immunodetection of gliadin in standard solutions, with a duration depending on the number of samples tested. More precisely, the use of the Q-TEM grid crystal compared to the S-QCM crystal leads to a great improvement in the limit of detection, at least in a ratio of 1000. In the case of Q-TEM grid crystal, the linearity of the sensor response and its sensitivity depend on the gold thickness deposed through the TEM grid. On the other hand, QCM-color crystal has been successfully used to detect gliadin in aqueous solution, showing an easy and inexpensive process of formation nanoparticles and nanoislands as effective as other methods of synthesizing nanoparticles. The observation of a positive shift of the resonance frequency (anti-Sauerbrey behavior) is in good agreement with published articles indicating that the interaction between the probe and the target is influenced by the metallic nanostructuration of the gold electrode. However, acoustic sensing using a QCM-color crystal was not applicable in the present format to test the content of real food products. In this case, additional studies are needed to optimize the process of extracting gliadin from real samples. Therefore, a continuous washing step instead of a drop collection protocol could be an elegant solution to reduce non-specific adsorptions on the gold electrode of QCM crystal.
